# The Prognostic Factors Affecting the Survival of Kurdistan Province COVID-19 Patients: A Cross-sectional Study From February to May 2020

**DOI:** 10.34172/ijhpm.2020.155

**Published:** 2020-08-22

**Authors:** Eghbal Zandkarimi, Ghobad Moradi, Behzad Mohsenpour

**Affiliations:** ^1^Kurdistan University of Medical Sciences, Sanandaj, Iran.; ^2^Department of Epidemiology and Biostatistics, Faculty of Medicine, Kurdistan University of Medical Sciences, Sanandaj, Iran.; ^3^Department of Infectious Disease, Liver and Digestive Research Center, Research Institute for Health Development, Kurdistan University of Medical Sciences, Sanandaj, Iran.

**Keywords:** COVID-19, Chronic Lung Disease, Chronic Kidney Disease, Coronary Heart Disease, Cox Proportional Hazard

## Abstract

**Background:** Coronavirus disease 2019 (COVID-19) is a new viral disease and in a short period of time, the world has been affected in various economic, social, and health aspects. This disease has a high rate of transmission and mortality. The aim of this study is to investigate the factors affecting the survival of COVID-19 patients in Kurdistan province.

**Methods:** In this retrospective study, the data including demographic features and the patient’s clinical background in terms of co-morbidities such as diabetes, cancer, chronic lung disease (CLD), coronary heart disease (CHD), chronic kidney disease (CKD) and weak immune system (WIS) were extracted from electronic medical records. We use Cox’s regression proportional hazard (PH) to model.

**Results:** In this study, out of 1831 patients, 1019 were males (55.7%) and 812 were females (44.3%) with an average age of 52.74 ± 22.16 years. For survival analysis, data from people infected with COVID-19 who died or were still being treated were used. According to Cox’s regression analysis, age variables (hazard ratio [HR]: 1.03, CI: 1.02-1.04), patients with a history of diabetes (HR: 2.16, CI: 1.38-3.38), cancer (HR: 3.57, CI: 1.82-7.02), CLD (HR: 2.21, CI: 1.22-4) and CHD (HR: 2.20, CI: 1.57-3.09) were significant and affected the hazard of death in patients with COVID-19 and assuming that the other variables in the model are constant, the hazard of death increases by 3% by increasing one unit (year), and the hazard of death in COVID-19 patients with CHD, diabetes, cancer, CLD is 2.16, 3.57, 2.2 and 2.21, respectively.

**Conclusion: **According to findings, it is necessary to evaluate the prevalence of COVID-19 in patients with CLD, diabetes, cancer, CHD, and elder, as patients with these characteristics may face a greater risk of death. Therefore, we suggest that elders and people with those underlying illnesses need to be under active surveillance and screened frequently.

## Background

**Key Messages**

Implications for policy makers

- Based on the findings of this study, the risk of death is higher in coronavirus disease 2019 (COVID-19) patients with underlying diseases such as cancer, coronary heart disease (CHD), diabetes, chronic lung disease (CLD), and the elderly.
- The prevalence of COVID-19 in people with underlying diseases and elders should be evaluated frequently.
- The COVID-19 patients at high risk of death should be kept away from silent carriers and be under surveillance and screened frequently.

Implications for the public

According to the results obtained of this study, in coronavirus disease 2019 (COVID-19) patients, the variables age, patients with a history of diabetes, cancer, chronic lung disease (CLD) and coronary heart disease (CHD) were significant and these factors affect the hazard of death in COVID-19 patients, so that, the hazard of death in the COVID-19 patients’ increases by 3% by increasing one year of age, for example, the risk of death for a 70-year-old is 3% higher than for a 69-year-old, also, the hazard of death in the COVID-19 patients’ with CHD, diabetes, cancer, CLD is 2.16, 3.57, 2.2, and 2.21 times more than COVID-19 patients without these underlying diseases, respectively.

Coronavirus disease 2019 (COVID-19) is a new viral disease with the animal origin and most likely transmitted from animal to human. The first cases of the disease were identified on December 8, 2019, with acute respiratory symptoms in Wuhan, Hubei province, China.^1^ This disease has a high rate of transmission from one person to another^2,3^ and from December 2019 to 16 July 2020, more than 14 million people have been reported with the virus, of which more than 600 000 have died from complications of this disease. Although the mortality rate for COVID-19 (6.8%)^4^ is relatively low, due to the high rate of pathogenicity of this virus, this percentage of death will be very high compared to other diseases. Iran, like many other countries in the world, became involved in this pandemic, so that, from February 20, 2020, to Jul 17, 2020, 273 656 total cases were identified, of them, 14 188 died.^4^ COVID-19 is an unknown disease and humans have little information about disease transmission methods, high-risk groups, etc, but in a small number of studies, it has been shown that COVID-19 patients with chronic and underlying diseases such as diabetes,^2,5^ chronic kidney disease (CKD),^6^ coronary heart disease (CHD),^7^ hypertension,^2^ cancer,^3^ and chronic lung disease (CLD)^2^ have a high risk of death. According to what has been said, COVID-19 disease has a high pathogenicity rate, and the question now is whether the risk of death is the same among COVID-19 patients? Are death rates the same among age groups, gender, and people with other underlying diseases? If the risk of death is not the same, what is the rate of this risk in different groups? Due to the fact that the world has been affected by this disease healthily and a comprehensive study (in Iran or abroad) have not been conducted to identify the high-risk group among COVID-19 patients, therefore, the aim of this study is to identify factors affecting on survival in COVID-19 patients.

## Objectives

 Here, we use the information of identified COVID-19 patients, with laboratory-confirmed, which hospitalized in Kurdistan province located in western Iran.

## Methods

###  Study Design and Definitions

 This study is retrospective. We use the information of Kurdistan province COVID-19 patients, western Iran, (identified by 16-hour centers) admitted to hospitals (16 hospitals) or under treatment at home. In Iran, in order to reduce the burden of hospital visits, centers called 16-hour have been set up, and people with symptoms such as cough, fever, etc are referred to these centers and examined. In these centers, samples are taken from the pharyngeal or nasal and sent to the Central Laboratory for examination. In this study, those who identified as COVID-19 between February 22, 2020, and May 18, 2020, were included in our study. According to guidelines published by the WHO and CDC,^8^ subjects with symptoms including fever, dry cough, vomiting, and diarrhea are suspected of being infected with the COVID-19, and patients with multiple comorbidities are prone to severe infection.^8^ In this study, suspicious cases were clinically diagnosed with reverse transcription–polymerase chain reaction (RT-PCR). In this study, the effect of underlying diseases on the survival of COVID-19 patients is investigated. We mean of WIS, people with underlying diseases such as HIV, malnutrition, and viral hepatitis, also we mean of CLD the following diseases: (1) Asthma, (2) Chronic obstructive pulmonary disease, and (3) chronic pneumonia.

 In this study, all types of cancer (include both solid tumors and hematological malignancies) were analyzed and we mean diabetes both types I and II.

###  Data Collection 

 Clinical retrospective data including demographic variables, treatment status, the patient’s clinical background in terms of comorbidities diseases such as diabetes, cancer, CLD, CHD, CKD, and weak immune system (WIS) were extracted from electronic medical records. The data is taken from the COVID-19 information registration portal of the disease prevention unit of the Vice-Chancellor for the health of Kurdistan University of Medical Sciences.

###  Statistical Analyses

 The gender (male 1: female 0), age (continuously), patients with CLD (yes 1: no 0), patients with CHD (yes 1: no 0) and patients with CKD (yes 1: no 0), patients with cancer diseases (yes 1: no 0) and patients with WIS diseases (yes 1: no 0) there were considered as independent variables. We used the chi-square test to compare the differences between the 3 groups (Non-survivor, recovered, and under treatment). Cox’s regression proportional hazard (PH) was used to model and we use the goodness of fit based on the Schoenfeld residuals to test the PH assumption. We used the Kaplan-Meier curve and log-rank test to compare survival times between groups. The time interval between identifying individuals as COVID-19 and the time of death or the end of the study was considered as the time of survival or censorship (response variable) and if the individual died during the study, he/she has event status and gets code 1 and if the individual survive during and not recover, he/she has censor status and gets code 0. Statistical analysis was used statistical software R version 3.5.

## Results

 In Table 1 continuous and categorical variables were presented as n (%). The study used data from 1831 patients, including 1019 (55.7%) men and 812 (44.3%) women, with an average age of 52.74 ± 22.16 years. Of the 1831 patients (COVID-19), 142 (7.8%) patients died, 943 (51.5%) recovered, and 746 (40.7%) under treatment (under home care or hospitalization). According to Table 1, subjects with CHD (20.3%), diabetes (9.3%), and CLD (6%) had a large frequency in the subjects with underlying diseases. In the investigated sample, dry cough (57.7%), fever (33.7%), and sore throat (16.4%) were the most common symptoms. We used the chi-square test to compare the differences between the three groups (Non-survivor, recovered, and under treatment). In the three groups mentioned above, there was a significant difference between diabetes (*P* < .001), CHD (*P* < .001), and patients with cancer diseases (*P* < .001). According to Table 1, among COVID-19 patients that were died, patients with a history of CHD (14.3%), diabetic patients (13.5%) and patients with a history of CLD (10.9%) have the highest frequency of death.

**Table 1:** Frequency, Relative Frequency and Chi-square Test of Demographic Variables and Clinical Features in Patients With COVID-19 in Kurdistan Province in Western Iran

**Variable**	**Non-survivor** **142 (7.8%)**	**Recovered** **943 (51.5%)**	**Under treatment** **746 (40.7%)**	**Total** **1831**	***P*** ** Value**
Age (y)	66.25 ± 15.78	50.70 ± 21.43	52.75 ± 20.23	52.74 ± 22.16	**-**
Gender					
Female (RC)	45 (5.5%)	423 (52.1%)	344 (42.4%)	812 (44.3%)	.006
Male	97 (9.5%)	520 (51.0%)	402 (39.5%)	1019 (55.7%)	
Disease history					
Diabetes	23 (13.5%)	99 (58.2%)	48 (28.2%)	170 (9.3%)	<.001
WIS	1 (12.5%)	5 (62.5%)	2 (25.0%)	8 (0.8%)	.634
CHD	53 (14.3%)	171 (46.1%)	147 (39.6%)	371 (20.3%)	<.001
CLD	12 (10.9%)	60 (54.5%)	38 (34.5%)	110 (6.1%)	.245
CKD	5 (10.4%)	22 (45.8%)	21 (43.8%)	48 (2.6%)	.648
Cancer	9 (28.1%)	15 (46.9%)	8 (25.0%)	32 (1.7%)	<.001
Symptoms					
Fever	61 (9.9%)	313 (50.7%)	243 (39.4%)	617 (33.7%)	.005
Dry cough	94 (8.9%)	524 (49.6%)	439 (41.5%)	1057 (57.7%)	.042
Diarrhea	1 (1.4%)	35 (47.9%)	37 (50.7%)	73 (4.1%)	.049
Vomit	9 (6.8%)	70 (52.6%)	54 (40.6%)	133 (7.3%)	.897
Headache	13 (6.5%)	118 (58.7%)	70 (34.8%)	201 (11.1%)	.355
Runny nose	4 (17.4%)	11 (47.8%)	8 (34.8%)	23 (1.3%)	.217
Chest-ache	11 (8.9%)	69 (56.1%)	43 (35.0%)	123 (6.7%)	.394
Sore throat	25 (8.3%)	140 (46.5%)	136 (45.2%)	301 (16.4%)	.163

Abbreviations: COVID-19, coronavirus disease 2019; WIS, weak immune system; CHD, coronary heart disease; CLD, chronic lung disease; CKD, chronic kidney disease; RC, reference category.

 In the following, the Kaplan-Meier curve was used to compare the estimated survival times of the groups. Then Cox’s PH model will then be used to estimate the hazard ratio (HR) and 95% confidence interval (CI). Kaplan-Meier’s curves for the demographic and clinical variables of patients with COVID-19 are shown in Figure.

**Figure:**
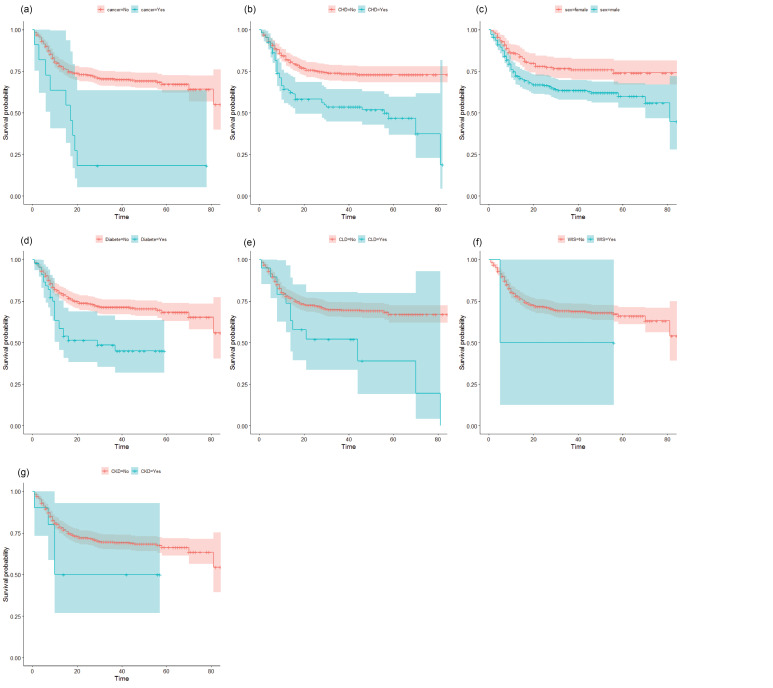
The Kaplan-Meier Curve for the Demographic and Clinical Variables in the COVID-19 Patients of Kurdistan province, Iran. (a) Cancer patients with COVID-19; (b) CHD patients with COVID-19; (c) Gender of COVID-19 patients; (d) Diabetes patients with COVID-19; (e) CLD patients with COVID-19; (f) WIS patients with COVID-19; (g) CKD patients with COVID-19. Abbreviations: COVID-19, coronavirus disease 2019; WIS, weak immune system; CHD, coronary heart disease; CLD, chronic lung disease; CKD, chronic kidney disease.

 According to the graphs and log-rank test (Table 2), WIS (*P* = .490), CKD (*P* = .130) were not statistically significant. According to the Kaplan-Meier curves (vertical distance), the probability of survival patients with COVID-19 in the subjects with CHD, diabetes, cancer and CLD are less than those without these diseases. Also, according to the horizontal distance of the Kaplan-Meier curves, patients with COVID-19 who had diabetes, CLD and CHD die sooner, and these patients (COVID-19) are less likely to survive than those without these underlying diseases (Figure).

**Table 2:** Results of Log-Rank Test in the COVID-19 Patients, Kurdistan Province, Iran

**Variable**	**Chi-square**	***df***	***P*** **Value** ^a^
Gender (female)	9.7	1	.002
Diabetic	13.8	1	<.001
WIS	0.5	1	.490
CHD	20.9	1	<.001
CLD	7.1	1	<.001
CKD	2.3	1	.130
Cancer	18.5	1	<.001

Abbreviations: COVID-19, coronavirus disease 2019; WIS, weak immune system; CHD, coronary heart disease; CLD, chronic lung disease; CKD, chronic kidney disease; df, degrees of freedom. ^a^A two-sided *P* <.050 was considered statistically significant.

 To analyze survival using the Cox model, we used the data of 888 COVID-19 patients who died and under treatment, and the information of the recovered people (n = 943) was not considered in this analysis. Before fitting the Cox model of PHs to survival data, we used the goodness of fit method to examine the hypothesis of PH. The results of this test are shown in Table 3, and according to these results, the PH assumption is established and the Cox PH model can be used.

**Table 3:** Results of the Test of the PH Assumption

**Variable**	**Chi-square**	***df***	***P*** ** Value**
Age	0.12	1	.730
Gender (ref: female)	0.17	1	.680
Diabetic	0.66	1	.420
WIS	0.72	1	.401
CHD	0.69	1	.401
CLD	2.64	1	.101
CKD	0.01	1	.980
Cancer	2.21	1	.140
Global test	6.75	1	.560

Abbreviations: PH, proportional hazard; WIS, weak immune system; CHD, coronary heart disease; CLD, chronic lung disease; CKD, chronic kidney disease; df, degrees of freedom. A two-sided *P* <.050 was considered statistically significant.

Table 4 shows the hazard (risk) ratio, 95% CI, and the probability value of demographic characteristics and underlying diseases. Cox’s regression PH was used to model, and according to the results obtained in COVID-19 patients the variables including age (HR: 1.03, CI: 1.02-1.04), patients with a history of diabetes (HR: 2.16, CI: 1.38-3.38), cancer (HR: 3.57, CI: 1.82-7.02), CLD (HR: 2.21, CI: 1.22-4) and CHD (HR: 2.20, CI: 1.57-3.09) were significant. In the COVID-19 patients, assuming that the other variables in the model are constant, the hazard of death increases by 3% by increasing one unit (year), and the hazard of death in COVID-19 patients with CHD, diabetes, cancer, CLD is 2.16, 3.57, 2.2 and 2.21, respectively.

**Table 4:** Results of Cox PH Model in the COVID-19 Patients, Kurdistan Province, Iran

**Variable**	**Survival Median**	**HR**	**95% CI**		***P*** **Value** ^a^
			**Lower**	**Upper**	
Age	-	1.03	1.02	1.04	<.001
Gender (RC: female)	81	1.73	0.51	2.47	.061
Diabetic	29	2.16	1.38	3.38	<.001
WIS	5	2.08	0.29	14.93	.460
CHD	56	2.20	1.57	3.09	<.001
CLD	44	2.21	1.22	4.01	.008
CKD	10	1.97	0.81	4.08	.140
Cancer	17	3.57	1.82	7.02	<.001

Abbreviations: PH, proportional hazard; COVID-19, coronavirus disease 2019; WIS, weak immune system; CHD, coronary heart disease; CLD, chronic lung disease; CKD, chronic kidney disease; HR, hazard ratio; RC, reference category. ^a^A two-sided *P* <.050 was considered statistically significant.

## Discussion

 COVID-19 belongs to the coronavirus family, in which 6 mutations have been identified so far.^9^ In the coronavirus family, severe acute respiratory syndrome (SARS) and Middle East respiratory syndrome (MERS) caused plagues and large numbers of deaths.^10^ This retrospective study identified several prognostic factors for death in COVID-19 patients in Kurdistan province. In particular, having a history of CHD, cancer, CLD, diabetes, and older age were associated with higher risk of death. The proportion of diseases of CHD, CLD, cancer, WIS, and diabetes were, 20.3%, 6%, 1.7%, 0.8% and 9.3%, respectively. Some of our findings are consistent with previous studies. This study confirmed that increased age increased the risk of death in patients with COVID-19 and for every 1 year of age, the risk of death increases by 3%, assuming that other variables are constant. In previous studies, older age has been reported as an important prognostic factor of mortality in SARS and MERS.^11-14^ Several studies have confirmed that increasing age in patients with COVID-19 is associated with death.^2,8,15^ The current study confirmed that the risk of death of COVID-19 in diabetic patients 2.16 times than a patient without diabetes. In some studies, diabetes has been reported as an important prognostic factor of mortality in SARS, influenza A (H1N1), and MERS.^16-18^ A meta-analysis confirmed that diabetic patients with COVID-19 infection were more likely to develop severe/intensive care unit cases.^9^ A recent study from Italy showed more than two-thirds of those who died by COVID-19 had diabetes.^19^ The study confirmed that the risk of death from COVID-19 in patients with CLD was 2.21 times that of patients without CLD. The study conducted by Yang et al confirmed that patients infected with COVID-19 who had chronic respiratory disease had a higher chance of increasing the infection.^20^ The study confirmed that the risk of death from COVID-19 in CHD patients was 2.2 times that of CHD-free patients. The study conducted by Zhou et al confirmed that CHD associated with fatal outcomes of COVID-19.^2^ The study confirmed that the risk of death from COVID-19 in cancer patients was 3.57 times that of patients without cancer. The study conducted by Zhang et al confirmed that patients infected with COVID-19 who had cancer disease had a higher chance of death.^3^ The study conducted by He et al confirmed that patients with hematological cancers had a similar rate of COVID-19 compared to normal healthcare providers, but that these patients had more severe disease and higher mortality rates.^21^ One of the strengths of this study was access to suitable sample size (1831 people) of different age and sex categories so that the results of this study can be generalized to the whole community but one of the weaknesses of this study is that the lack of access to some information (eg, socioeconomic status of patients) may affect the results and it is maybe to find a significant relationship between the survival of individuals with these variables, another weakness of this study is that we used data of those who were diagnosed by PCR and did not use data for the clinically-confirmed cases (both incidence and death), that failure to consider this group of people may lead to selection bias. Finally, based on the findings of this study, it is necessary to evaluate the prevalence of COVID-19 in patients with cancer, CHD, diabetes and CLD, and the elderly, so that, COVID-19 patients with these characteristics may face a greater risk of death. Therefore necessary that older people and people with underlying illnesses need to be surveillance and screened frequently.

## Acknowledgements

 We would like to thank the disease prevention unit of the Vice-Chancellor for the health of Kurdistan University of Medical Sciences for providing us with the study data.

## Ethical issues

 This study was approved by the Ethics Committee of the Kurdistan University of Medical Sciences Vice Chancellor for Research (No. IR.MUK.REC.1399/018).

## Competing interests

 Authors declare that they have no competing interests.

## Authors’ contributions

 The study was designed and analyzed by EZ. GM and BM contributed to the interpretation of the results and the writing of the article.

## Funding

 The study was funded by Vice-chancellor for Research and technology, Kurdistan University of Medical Sciences (No. IR.MUK.REC.1399/018).

## Authors’ affiliations

^1^Kurdistan University of Medical Sciences, Sanandaj, Iran. ^2^Department of Epidemiology and Biostatistics, Faculty of Medicine, Kurdistan University of Medical Sciences, Sanandaj, Iran. ^3^Department of Infectious Disease, Liver and Digestive Research Center, Research Institute for Health Development, Kurdistan University of Medical Sciences, Sanandaj, Iran.
